# On the Adaptability of Recurrent Neural Networks for Real-Time Jazz Improvisation Accompaniment

**DOI:** 10.3389/frai.2020.508727

**Published:** 2021-02-12

**Authors:** Kosmas Kritsis, Theatina Kylafi, Maximos Kaliakatsos-Papakostas, Aggelos Pikrakis, Vassilis Katsouros

**Affiliations:** ^1^ Department of Informatics, University of Piraeus, Piraeus, Greece; ^2^ Institute for Language and Speech Processing, Athena Research Center, Athens, Greece; ^3^ Department of Informatics and Telecommunications, National and Kapodistrian University of Athens, Athens, Greece

**Keywords:** automatic accompaniment system, music generative system, real-time music interaction, music improvisation, machine learning, long short-term memory

## Abstract

Jazz improvisation on a given lead sheet with chords is an interesting scenario for studying the behaviour of artificial agents when they collaborate with humans. Specifically in jazz improvisation, the role of the accompanist is crucial for reflecting the harmonic and metric characteristics of a jazz standard, while identifying in real-time the intentions of the soloist and adapt the accompanying performance parameters accordingly. This paper presents a study on a basic implementation of an artificial jazz accompanist, which provides accompanying chord voicings to a human soloist that is conditioned by the soloing input and the harmonic and metric information provided in a lead sheet chart. The model of the artificial agent includes a separate model for predicting the intentions of the human soloist, towards providing proper accompaniment to the human performer in real-time. Simple implementations of Recurrent Neural Networks are employed both for modeling the predictions of the artificial agent and for modeling the expectations of human intention. A publicly available dataset is modified with a probabilistic refinement process for including all the necessary information for the task at hand and test-case compositions on two jazz standards show the ability of the system to comply with the harmonic constraints within the chart. Furthermore, the system is indicated to be able to provide varying output with different soloing conditions, while there is no significant sacrifice of “musicality” in generated music, as shown in subjective evaluations. Some important limitations that need to be addressed for obtaining more informative results on the potential of the examined approach are also discussed.

## Introduction

The use of automatic systems for generating music is a captivating vision and a multidisciplinary research problem studied for decades. The diversity of music generative systems relies on their different objectives and the musical content that they produce, such as chord progressions, melody generation, accompaniment arrangements and counterpoints (Briot et al., 2019). Already from the late 1950s and early 1960s, composers such as Lejaren A. Hiller (Hiller Jr and Isaacson, 1957) and Iannis Xenakis (Xenakis, 1963) explored stochastic models for algorithmic music generation.

With the recent advances in the computational capabilities of modern computers, there is an exploding tendency of generative system proposals, incorporating complex artificial neural network architectures as a technical foundation. Conditional generative models based on Generative Adversarial Networks (GANs) have been used to combine unpaired lead sheet and MIDI datasets for generating lead sheet arrangements. The lead sheet arrangement can be defined as the process that receives a lead sheet as input and outputs piano-rolls of a number of instruments to accompany the melody of a given lead sheet. Liu and Yang (2018) proposed an architecture that comprises three stages (lead sheet generation, feature extraction and arrangement generation) in order to generate eight-bar phrases of lead sheets and their arrangement. The feature extraction stage is responsible to compute symbolic-domain harmonic features from the given lead sheet in order to condition the generation of the arrangement. Wang and Xia (2018) developed a framework for generating both lead melody and piano accompaniment arrangements of pop music. Specifically, they consider a chord progression as input and propose three phases for generating a structured melody with layered piano accompaniments. First, the harmony alternation model receives a given chord progression in order to transform it to a different one that fits better with a specified music style based on Hidden Markov Models (HMMs). Then, the melody generation model generates the lead melody and the layered accompaniment voices through seasonal ARMA (Autoregressive Moving Average) processes. The final phase implements the melody integration model which is responsible for integrating the melody voices together as the final piano accompaniment.

On the other hand, Recurrent Neural Networks (RNNs) are often used to generate sequences of musical content in a stepwise manner, where the network input is the previous note and output is considered the predicted note to occur on the following time interval (Mozer, 1994). In a similar manner, RNNs with Long Short-Term Memory (LSTM) cells have been utilized for generating blues style melodies conditioned by a given chord progression (Eck and Schmidhuber, 2002). By definition, LSTM-based models have the ability to correlate and capture the temporal context of a sequence, thus simulating the human cognitive abilities for predicting sequential information. Also, RNNs have proven efficacy on modelling complex musical structures such as polyphonic chorales. For instance, the DeepBach system was trained to generate four-part chorales in the style of J. S. Bach (Hadjeres et al., 2017). Generative systems can be also constrained by music theory rules via a reinforcement learning mechanism as it is demonstrated by Jaques et al. (2017). In addition to the music theory rules, Boulanger-Lewandowski et al. (2012) employed probabilistic harmonic and rhythmic rules, based on distribution estimators conditioned by a RNN that is trained to discover temporal dependencies from polyphonic music scores of varying complexity.

Other approaches take into account the chord progressions for providing longer musical structures. For instance, in the work of Choi et al. (2016), a text-based LSTM network is employed for capturing the relationships within text documents that contain symbols of chord progressions. Another example based on chord progressions is the JamBot system (Brunner et al., 2017) that generates music in two steps. The bottom network is a LSTM architecture that predicts a chord progression based on a chord “embedding,” while a second LSTM generates polyphonic music based on the predicted chord progression received from the bottom network. Nevertheless, this approach lacks the ability of modeling interactions within a polyphonic musical ensemble. In order to overcome this limitation, Chu et al. (2016) proposed a hierarchical architecture, where each level is a RNN that generates different accompaniments for the song. A monophonic melody is generated first, followed by the accompanying chords and drums.

In the scope of the Impro-Visor (Jazz Improvisation Advisor)^1^ project, Johnson et al. (2017) proposed a neural network architecture consisting of two LSTM-based sub-networks that jointly learn to predict a probability distribution over future notes conditioned on past notes in the melody. Additionally, researchers from the same laboratory developed the JazzGAN system (Trieu and Keller, 2018) that utilizes RNN-based GANs to improvise monophonic jazz melodies over given chord progressions. Their results indicated that the proposed system was capable to address frequent and diverse key changes, as well as unconventional and off-beat rhythms, while providing flexibility with off-chord notes. Other proposals incorporate music theory grammar in combination with LSTM neural networks to generate jazz music. For instance, Wang et al. (2019) extracted the interval, duration and note category information from jazz MIDI files and trained a LSTM model to learn the transition probabilities between notes. Then they take advantage of the music grammar in order to arrange and output the generated sequence of notes.

LSTM networks have been also tested for generating jazz music compositions constrained by a given performer’s style. In particular, De Prisco et al. (2017) developed a three staged generative system, consisting of a One-Class Support Vector Machine (OCSVM) for learning the performing style of a specific jazz musician, an LSTM network to generate patterns relevant to the learned style and a splicing system to compose melodic lines in the given style. Splicing systems are formal models for generating languages (sets of words), inspired by a recombinant behavior of DNA (De Felice et al., 2015). A music splicing composer requires to define an alphabet, an initial set and a set of rules. Another example of a complex system that utilizes LSTM networks for learning statistical correlations between instruments within a jazz piano trio ensemble (piano, bass, drums) was proposed by Hori et al. (2017). They trained a LSTM architecture to learn the relationship between the musical features of the piano performance that is applied on top of a Hidden Markov Model (HMM), which is responsible to segment the bass and drums performance feature spaces. Overall the system is capable to generate coherent rhythmic patters and bass melodies as accompaniments to a piano solo input. However the authors specify that their model can be further improved due to the lack of available jazz datasets. To this regard, Hung et al. (2019) employed transfer learning techniques aiming to solve the problem of jazz data insufficiency. They proposed a Bidirectional Gated Recurrent Unit (BGRU) Variational Autoencoder (VAE) generative model trained on a dataset of unspecified genres as source and a Jazz-only dataset as target.

It is worth noting that only a few projects experiment with real-time creative scenarios where a human improviser is accompanied by an automatic agent without any musical constraints. To this end, Kaliakatsos-Papakostas et al. (2012) proposed an accompaniment system that employs Differential Evolution and Genetic Algorithms for producing the accompanying music. Another approach to real-time music generation for jazz improvisation that was proposed by Hutchings and McCormack (2017), implements a composite system with an LSTM-based melody agent, which was trained on chord progressions of jazz “standard” compositions and a rule-based harmony agent that manipulates precomposed melodies for improvising new themes and variations. The composition flow between the agents is controlled by a rating system that rewards harmonic consistency and melodic continuity.

Aim of this paper is to examine the characteristics of musical accompaniment that an artificial agent can provide in real-time to a human improviser, in a setting similar to typical forms of jazz improvisation, i.e. under the constraints of previously agreed upon harmonic sequence and metrical structure. Software tools and methods that are able to generate “static” accompaniment to human soloists, exist for a long time (Ferguson, 2005). The paradigm discussed in this paper includes “spontaneous” alterations in accompaniment responses of an artificial agent both in terms of rhythm and harmony, based on the improvisation of a human soloist. The algorithmic cornerstone of the examined approach relies on LSTM RNNs architectures. The motivation for pursuing and studying such an approach in modeling human-machine improvisation and the reasons for choosing to examine basic deep learning neural networks as an algorithmic backbone is analysed in the following section.

## Motivation, Research Questions and Contribution

In music, “masterful” violation of anticipation has been identified as key component for the emergence of emotion, meaning, concepts and overall interest (Huron, 2006). Furthermore, anticipation is shaped by the exposure to stimuli with common characteristics, a fact that induces relations between fundamental mechanisms of music understanding and statistical learning (Huron, 2006). The basic principles of jazz improvisation evolve around the violation of expectation, with improvising musicians constantly attempting to introduce meaningful novelty in the way they express themselves and communicate with other musicians in real-time. Therefore, jazz improvisation could be described as an exemplar for studying the core-mechanism of music cognition: interplay between anticipation and violation thereof.

Communication between improvising musicians is a key-point for achieving interesting and meaningful improvisations. In jazz improvisation, specifically, the role of each musician is manifold; the most prominent characteristics of the role of each musician, according to how they relate with the study at hand, can be summarised as follows:

1. *Preserve harmonic and rhythmic characteristics of a piece.* Typical jazz improvisation incorporates a standard jazz melody with a fixed harmonic description in a fixed metric structure. These components, however, are expected to be creatively altered by improvising musicians (usually not the metric structure though), towards creating meaningful violations of anticipation on the overall harmonic and rhythmic domain. For instance, chord substitutions are usual, either by introducing chords that include alternate voicings, extensions or even by including new chords altogether (e.g. tritone substitution).
2. *Express original ideas.* Violation of harmonic/rhythmic expectations is expected to come “with a reason.” A common approach for soloists to attempt to build new musical phrases when improvising, is by creatively modifying and combining “standard” jazz licks, a fact that helps towards building and violating anticipation. Jazz licks in the (muscle) memory of the soloist are products of statistical learning, built through practicing and listening multiple jazz pieces, excerpts and phrases.
3. *Communicate musically with the improvisation/accompaniment of other musicians.* In a broad sense, the role of the accompanist is to highlight musical choices of the soloist, or, even further, understand the intentions of the soloist and improvise accompaniments accordingly. Therefore, communication, on the side of the accompanist, includes predicting the intentions of the soloist and preparing the response in a timely manner, given that proper accompaniment needs to be provided concurrently with the solo. Jazz musicians, as musicians in any other field, develop a common perception that, in the examined case, can be described as the integration of a “similar” statistical model both in the soloist and the accompanist; this model allows the accompanist to roughly predict the imminent soloist choices during improvisation.

To this end, an artificial agent that is able to perform *basic* musical accompaniment in real-time under the aforementioned setting needs to have: 1) the ability to comply with harmonic and metrical constraints set by an input chart; 2) a model of anticipating for imminent actions of the human soloist; 3) a dictionary of accompanying voicings for given chords that is rich enough for producing diverse/interesting accompaniment; and 4) the ability to “adapt” its playing style (both in terms of voicings and rhythm) to the anticipated choices of the human soloist. Since the problem description incorporates statistical learning and given the fact that deep neural networks have exhibited impressive capabilities in capturing the prominent statistical behaviour in large amounts of training data, this study examines the incorporation of such machine learning tools for the task at hand. Therefore, the research questions revolve around the suitability of deep learning methods for the described improvisation setting, under the methodological framework that is presented in *Materials and Methods*. These questions are formulated as follows:

- Is the presented framework able to capture “static” harmonic information of a given chart in a setting of dynamic constraints (changing playing style of the human soloist)?
- To what extent is the proposed system responding to dynamic components introduced by the human agent?
- Is the examined setup suitable for real-time performance, both in terms of robustness and computational feasibility?

Recent advances in deep learning include the development of systems that are able to generate music that adapts to pre-configured constraints. In general terms, such systems either compose music *sequentially* or *non-sequentially*. In sequential systems (e.g. as the one presented by Makris et al. (2019)), the decision for each note depends only on previous notes, with additional potential constraints. In non-sequential systems (e.g., as Deep Bach; Hadjeres et al. (2017)), new notes are inserted by sampling, forming “dynamic” constraints for notes that are inserted later on, regardless of time precedence – i.e. notes at the end of the piece could be inserted at an earlier stage than notes earlier in the piece, depending on randomly sampled priorities. In one sense, a system that is able to perform real-time accompaniment, as described in the presented study, needs to be able to both compose sequentially (since time moves forward while performing) and comply with constraints that change as the composition is constructed (since the human soloist is expected to violate the expectations reflected by the solo predictive model).

The main contribution of this paper is that it studies the characteristics of a complex, multi-layered neural network where both static and dynamic components are combined for preforming predictions. The real-time improvisation setup discussed herein offers a well-defined platform of experimentation with potential interest for real-world applicability and clearly defined research questions.

## Materials and Methods

The proposed system provides real-time accompaniment to a human musician, based on a given harmonic description of lead sheet chord symbols. The role of the system is to reflect harmonic information as given in the lead sheet and also interpret this information with variability, responding to the predicted implied harmonic variability of a human solo. To this end, data need to include information about: 1) metric structure, for letting the system become aware of measure changes; 2) lead sheet information, for learning to comply with given lead sheet chords; 3) a human solo channel, for learning to respond to what the human soloist is expected to play; and 4) an accompaniment channel, for learning to play proper accompaniment chords/voicings over the given lead sheet chords. Up to our knowledge, such a dataset containing all the aforementioned properties is missing from the research community. To this end, *Data Preparation* describes the processes for constructing a dataset by starting off with an initial dataset collected from online resources that covers most of the requirements. Afterwards, we present the proposed system that incorporates two layers of information processing: the first for predicting the imminent steps of the human performance and the second for integrating this prediction along with other static constraints (i.e. metric and lead sheet information) for making the final chord accompaniment prediction.

### Data Preparation

The initial dataset^2^ (Liu and Yang, 2018) contains all necessary information about the pieces, including tempo, beat, melody and the chords on a lead sheet. It should be noted that only lead sheet information is included in this dataset without actual notation of the accompaniment chords. In order to address this issue we performed a harmonic enrichment procedure that is discribed in detail later in this section. Furthermore, the beat information indicates the start of a measure. A single time step corresponds to the 1/24 of a quarter note, a time resolution which is fine enough to even represent rhythm values of sixty-fourth triples. The melody and the accompanying chords are represented as 128-key piano rolls with the aforementioned time resolution, where each active note at each time instance is annotated with the respective velocity value. With this representation however, the information about a note repetition is potentially obscured. For instance, there is no differentiation between a single note/chord of a quarter duration (24 time steps) and two successive notes/chords with a duration of an eighth per note (12 time steps). A time resolution reduction from 24 steps per beat (quarter) to two steps per beat was performed, such that each time step was represented by 1/2 of a quarter note, which is an eighth note. In other words, from each beat (24 time steps) we only kept the melodic information of the first and 13th time step, by splitting each quarter (24 time steps) in half. Thus keeping only the first of each of the two subsets of time instances (12 time steps).

In order to construct a suitable and compact representation of chord information in the form of a jazz standard lead sheet, we use the information extracted from the accompanying chords channel of the initial dataset. Specifically, instead of keeping the velocity values of the chord notes and their MIDI numbers, we only kept the pitch class of their root, as well as the type of those chords, by using ready-made functions from the MIT Music21 *Python* library^3^, which contains a set of functions for computer-aided musicology. Moreover, we chose to represent the jazz standard chord information as a binary vector of size 15, where the first 12 bits represent the root pitch class information, while the remaining 3 bits represent major/minor third, perfect/augmented/diminished fifth and major/minor seventh respectively. The reason for performing such an abstraction for representing chord information on the lead sheet is motivated by the fact that jazz musicians need a fundamental description of harmony, which they can manipulate/alter in a creative manner. The employed scheme allows for basic chord types to be represented, e.g., major/minor triads, dominant seventh, major seventh, (half) diminished and augmented.

As mentioned earlier, the initially obtained dataset includes information only about lead sheet chords, without specific notation of actual accompanying chords. Hence we constructed the actual accompaniment chords algorithmically by applying a basic “harmonic enrichment” process, where the lead sheet chords are transcribed into actual accompanying chords with different inversions and diverse rhythmic patterns. The enrichment process begins by assigning accompaniment chords to positions of lead sheet chord symbols. After that, inverted chords are probabilistically inserted after the initially placed chords. The probability of chord insertion at a specific position on the score depends on the time passed without a chord event (the more the time, the higher the probability) and whether there is a melodic note event (melody notes increase the probability for chord insertion). Aim of this process is to introduce rudimentary variability in the accompaniment channel, based on the lead sheet chord symbols and the melodic rhythm.

Since the melody channel is monophonic, the 128-sized vector representation of each note in the melody channel is flattened to its single non-zero value (the actual MIDI number of that note). For the accompaniment channel, i.e. the actual notes that the system is intended to learn, a dictionary of all the unique chords in the training set is created and each chord is represented by its index in the dictionary. Practically, the “flattened” values for both the melody and the accompaniment parts allow us to apply one-hot representation of the respective data streams. Before the harmonic enrichment process, the initial dictionary of the accompaniment chords incorporated 476 chord classes, while after the augmentation and before the transposition to all the possible 12 pitches we had 847 classes. Finally, after all the data preparation procedure, including the augmentation and transposition processes, we ended up having 2677 unique accompanying chord classes.

### System Architecture and Real-time Considerations

**FIGURE 1:**
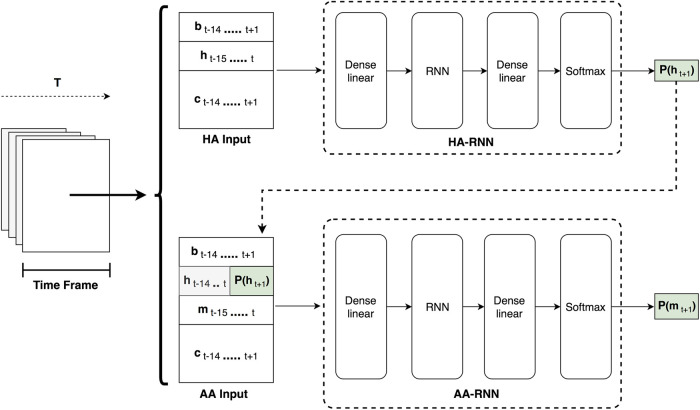
A detailed overview of the proposed system architecture. Consecutive overlapping time frames are processed by the two sub-systems. The HA-RNN predicts the soloist melody that is later used by the AA-RNN for predicting the accompaniment chords for the following time step.

As it is already mentioned, the generated accompaniment part should be related to the soloist’s intentions on the future melody notes to be played. To this regard, the proposed system architecture depicted in Figure 1, consists of two sub-systems, namely the Human Agent RNN (HA-RNN) and the Artificial Agent RNN (AA-RNN), that rely on the effectiveness of the LSTM recurrent neural network (RNN) for modeling sequential information.

The overall system receives as input successively overlapping windows comprising 16 time steps, representing events within a time resolution of eighth notes. The window slides one step/eighth note at each iteration, which occurs in every eighth successively. Information for each time step includes:

- The metric information ($b_{t}$).
- The soloist’s melodic/solo part ($h_{t}$).
- The accompaniment chords that are expected to be learned from the system ($m_{t}$).
- The chord information in the abstract lead sheet style described in *Data Preparation* ($c_{t}$).

Since the HA-RNN is responsible for predicting the solo melody of the following time step ($h_{t+1}$), it excludes the accompaniment channel from its input, while having the beat and chord information channels one eighth ahead from the current melody. On the other hand, the AA-RNN takes under account all the information channels, in addition to the predicted $P\left(h_{t+1}\right)$ of the HA-RNN, in order to anticipate the accompaniment chord for the future eighth ($m_{t+1}$). Both agents at their core, implement a similar neural network architecture. Firstly, the input time frame is processed by the bottom “Dense linear” (fully connected) layer, where it gets embedded to a fixed size dimension through a linear transformation. Next, the embedded output is further encoded into a latent space through the LSTM RNN layer. Then, the top “Dense linear” layer receives the encoded LSTM output and applies a linear transform to a space with a dimensionality equal to the number of the target classes. Finally the output of the top fully connected layer passes through a softmax function, resulting to a probability distribution for the target classes (P(h) and P(m)). The final prediction is the class with the highest probability.

As a proof-of-concept, we trained a basic system with batches of 128 samples. The embedding dimension of the bottom fully connected layer was equal to the size of the feature dimension of the input frame, whilst the RNN layer contained 64 LSTM cells. We used the Adam optimisation algorithm (Kingma and Ba, 2014) for the minimization of the cross entropy cost function with a learning rate of 0.001. Both the HA-RNN and AA-RNN architectures were implemented using the TensorFlow 2.0 framework (Abadi et al., 2016) and trained for at least 1,200 epochs on a computer equipped with the NVIDIA Tesla K40c GPU, an Intel Core i7-5820K CPU at 3.30 GHz and 32 GB DDR4 RAM at 2133 Mhz. With the aforementioned experimental setup, we observed that the average time of the overall system to predict an accompaniment chord was around 0.66 ms (0.31 ms for the HA in addition to 0.35 ms for the AA). This fact indicates the feasibility of the proposed system to be adopted in real-time applications, however a thorough evaluation of the real-time capabilities of the presented method needs to be examined as future work. In this regard, we developed a prototype web application based on MIDI.js and Tensorflow.js javascript libraries for testing the adaptability of the proposed model to the user’s soloing input in a real-time setting. The model implementation and training code of the LSTM models, as well as the real-time web interface are hosted on a GitHub repository^4^. Since the project continuously evolves, the online repository will be updated with future developments and improvements.

## Results

The results are oriented towards answering the research questions given in *Motivation, Research Questions and Contribution*, i.e. whether and to what extent is the system able to capture the harmonic lead sheet constraints, to what extent is the system influenced by different soloing styles and what are possible limitations for applying this approach in real-time settings with current technologies. To this end, two test jazz standards, “*All of Me*” and “*Au Privave*” are examined in different and diverse artificial improvisation settings, that simulate two extreme scenarios: where the human player 1) is not playing any note during the solo (consecutive occurrences of pause events) and 2**)** is playing random notes within two octaves (as a form of extremely complex improvisation). The responses of the system under these two settings for each piece are analysed for different epochs of training (randomly sampled across all training epochs), providing insights about how harmonic compliance is varied and how the existence of a solo affects system responses (adaptability) at different stages of training. Since technical limitations led to building a system with limited computational power (incorporating solely a single LSTM layer with few neurons for the artificial agent) and keeping time resolution to eight notes, getting useful feedback from musicians through exhaustive real-time experiments was not possible. In this regard, a preliminary empirical evaluation based on listening tests was conducted by comparing generated and original accompaniments. We maintain, however, that the results presented herein indicate that employing more sophisticated architectures for (at least) the part of the artificial agent would lead to a system that both adapts to the playing style of the user and preserves harmonic consistency according to the given lead sheet.

### Compliance with Lead Sheet Harmony

This section examines the ability of the system to play chords that correspond to the chord symbols on the lead sheet chart. This part of the study concerns the compliance with the basic harmonic guidelines provided by the chart and, therefore, comparison is presented on the level of pitch class sets (PC-sets). To this end, the lead sheet chart chords are translated to their corresponding pitch classes as well as the interpretations of the system. To obtain insight on how training epochs influence the harmonic compliance of the system, results are taken from an early and a late epoch of training (59 and 1,251). Table 1 (epoch 59) and Table 2 (epoch 1,251) show the chord symbols and the responses of the system in “All of me” when no solo (top) and random solo (bottom) was provided; similarly, Tables 3, 4 show the responses of the system in “Au Privave” without and with random solo.

**TABLE 1:** System interpretations of chart chords for “All of Me” without solo (top) and with random (bottom) solo at epoch 59, shown as pitch class sets.

No solo Chart chord	System interpretations				
[0, 4, 7, 11]	[0, 4, 7, 11] (80)	[0, 3, 8] (4)	[2, 7, 10] (12)	[0, 2, 4, 5, 7] (24)	
[0, 4, 7, 10]	[0, 5, 7, 10] (1)	[2, 6, 11] (1)	[2, 4, 6, 8] (4)	[2, 4, 8, 11] (1)	[0, 4, 7, 10] (25)
[2, 4, 8, 11]	[2, 6, 9, 11] (1)	[2, 4, 8, 11] (185)	[0, 4, 7] (4)	[0, 4, 7, 10] (18)	
[1, 4, 7, 9]	[1, 4, 7, 9] (168)	[2, 5, 9] (4)	[2, 7, 10] (4)		
[2, 5, 9]	[2, 5, 9] (128)				
[0, 4, 9]	[0, 4, 9] (64)				
[0, 2, 6, 9]	[0, 2, 6, 9] (64)				
[0, 2, 5, 9]	[1, 4, 7, 9] (8)	[0, 2, 5, 9] (72)			
[2, 5, 7, 11]	[2, 5, 7, 11] (79)				
[0, 5, 9]	[0, 5, 9] (32)				
[0, 5, 8]	[0, 5, 9] (4)	[0, 5, 8] (28)			
**Random solo** **Chart chord**	**System interpretations**			
[0, 4, 7, 11]	[4, 6, 8, 10, 11] (1)	[4, 6, 8, 11] (3)	[2, 6, 11] (3)	[0, 4, 7, 11] (80)	
		[0, 3, 8] (4)	[2, 7, 10] (12)	[0, 2, 4, 5, 7] (24)	
[0, 4, 7, 10]	[0, 4, 7, 10] (32)				
[2, 4, 8, 11]	[0, 4, 7] (4)	[2, 4, 8, 11] (186)	[0, 4, 7, 10] (18)		
[1, 4, 7, 9]	[1, 4, 7, 9] (168)	[2, 5, 9] (4)	[2, 7, 10] (4)		
[2, 5, 9]	[2, 5, 9] (128)				
[0, 4, 9]	[0, 4, 9] (64)				
[0, 2, 6, 9]	[0, 2, 6, 9] (64)				
[0, 2, 5, 9]	[1, 4, 7, 9] (8)	[0, 2, 5, 9] (72)			
[2, 5, 7, 11]	[2, 5, 7, 11] (79)				
[0, 5, 9]	[0, 5, 9] (32)				
[0, 5, 8]	[2, 5, 9] (4)	[0, 5, 8] (28)			

Numbers in parentheses show the total time steps that a system-generated PC-set occurs under the respective chart PC-set.

**TABLE 2:** System interpretations of chart chords for “All of Me” without solo (top) and with random (bottom) solo, shown as pitch class sets.

No solo Chart chord	System interpretations				
[0, 4, 7, 11]	[3, 5, 8, 11] (2)	[0, 2, 9, 10] (1)	[2, 4, 9] (1)	[1, 3, 10, 11] (1)	[1, 3, 6, 10] (1)
	[0, 3, 8] (1)	[2, 5, 10] (1)	[0, 4, 7, 11] (103)	[3, 7, 10] (13)	[0, 5, 9] (3)
[0, 4, 7, 10]	[0, 3, 8] (2)	[2, 5, 10] (1)	[0, 4, 7, 10] (23)	[2, 5, 7, 11] (3)	[2, 7, 11] (3)
[2, 4, 8, 11]	[2, 4, 8, 11] (162)	[4, 8, 11] (1)	[4, 7, 11] (4)	[0, 3, 5, 9] (1)	[3, 6, 8, 11] (1)
	[1, 4, 9] (1)	[1, 4, 7, 9] (12)	[1, 4, 6, 10] (3)	[2, 6, 8, 11] (3)	[2, 5, 9] (6)
	[0, 2, 5, 9] (3)	[0, 2, 6, 9] (3)			
[1, 4, 7, 9]	[1, 4, 7, 9] (124)	[2, 4, 7, 11] (9)	[1, 2, 6, 9] (11)	[2, 4, 8, 11] (7)	[1, 3, 10, 11] (4)
	[2, 6, 8, 11] (4)	[5, 8, 11] (3)	[2, 8, 11] (3)		
[2, 5, 9]	[2, 5, 7, 9] (15)	[2, 7, 10] (4)	[2, 5, 9] (94)	[1, 3, 10, 11] (3)	[1, 5, 8, 10] (3)
	[3, 7, 10] (6)				
[0, 4, 9]	[0, 4, 9] (12)	[3, 6, 10] (1)	[1, 6, 9] (1)	[4, 8, 11] (1)	[0, 2, 4, 9] (45)
	[2, 5, 9] (3)				
[0, 2, 6, 9]	[0, 2, 6, 9] (54)	[0, 4, 9] (5)	[0, 3, 6, 10] (1)	[0, 5, 8] (1)	[0, 3, 5, 8] (1)
	[2, 5, 10] (1)				
[0, 2, 5, 9]	[2, 5, 10] (13)	[0, 2, 5, 9] (36)	[0, 3, 7, 10] (2)	[3, 7, 10] (1)	[1, 3, 4, 11] (8)
	[0, 5, 8] (12)	[2, 4, 7, 11] (4)	[2, 5, 7, 10] (4)		
[2, 5, 7, 11]	[2, 5, 7, 11] (65)	[2, 3, 7, 10] (4)	[0, 3, 7] (4)	[0, 5, 8] (3)	[2, 5, 9, 10] (3)
[0, 5, 9]	[0, 4, 5, 9] (28)	[2, 5, 9] (4)			
[0, 5, 8]	[0, 5, 8] (28)	[3, 7, 10] (4)			
**Random solo** **Chart chord**	**System interpretations**				
[0, 4, 7, 11]	[3, 5, 8, 11] (1)	[1, 3, 6, 8] (2)	[2, 4, 9] (1)	[0, 4, 5, 9] (1)	[1, 3, 6, 10] (1)
	[2, 7, 10] (1)	[0, 5, 9] (5)	[0, 4, 7, 11] (86)	[3, 7, 10] (5)	[0, 2, 4, 5, 7] (14)
	[0, 4, 7] (2)	[2, 4, 5, 9] (1)	[1, 5, 8, 11] (1)	[2, 5, 10] (1)	[0, 2, 6, 9] (1)
	[0, 3, 6, 8] (1)	[1, 4, 9] (2)	[2, 5, 9, 10] (1)		
[0, 4, 7, 10]	[0, 4, 7, 10] (28)	[1, 5, 8] (1)	[0, 4, 5, 9] (1)	[2, 7, 11] (2)	
[2, 4, 8, 11]	[2, 4, 8, 11] (177)	[1, 4, 7, 9] (12)	[4, 8, 11] (2)	[4, 7, 11] (3)	[1, 4, 8, 11] (2)
	[1, 6, 9] (2)	[2, 4, 5, 9] (1)	[3, 6, 9, 11] (1)	[2, 4, 7, 11] (1)	
[1, 4, 7, 9]	[1, 4, 7, 9] (140)	[1, 2, 6, 9] (5)	[2, 4, 8, 11] (5)	[1, 4, 6, 10] (4)	[1, 6, 8, 11] (5)
	[0, 2, 6, 9] (6)	[2, 4, 7, 11] (4)	[2, 4, 5, 9] (1)		
[2, 5, 9]	[2, 5, 9] (95)	[2, 5, 7, 9] (30)	[2, 7, 10] (2)	[0, 4, 7] (1)	
[0, 4, 9]	[0, 2, 4, 9] (30)	[2, 5, 9] (2)	[0, 4, 9] (25)	[1, 3, 10, 11] (2)	[2, 4, 6, 7] (2)
	[4, 8, 11] (1)				
[0, 2, 6, 9]	[0, 2, 6, 9] (46)	[0, 4, 9] (8)	[1, 3, 10, 11] (2)	[2, 5, 7, 10] (2)	[0, 4, 7, 9] (2)
	[2, 7, 11] (2)				
[0, 2, 5, 9]	[0, 2, 5, 9] (49)	[1, 3, 10, 11] (3)	[0, 2, 5, 8] (2)	[0, 4, 7] (6)	[0, 1, 5, 8] (2)
	[2, 5, 7, 10] (6)	[3, 7, 10] (3)	[2, 6, 9] (2)	[0, 3, 5, 8] (2)	[2, 5, 10] (2)
	[0, 5, 8] (1)	[0, 2, 5, 7] (1)			
[2, 5, 7, 11]	[2, 5, 7, 11] (62)	[2, 5, 9, 10] (2)	[2, 5, 10] (8)	[2, 3, 7, 10] (1)	[2, 7, 11] (1)
	[5, 8, 11] (1)	[0, 4, 7, 10] (1)	[0, 2, 5, 9] (1)	[0, 4, 7] (1)	
[0, 5, 9]	[0, 4, 5, 9] (28)	[2, 5, 9] (4)			
[0, 5, 8]	[0, 5, 8] (28)	[3, 7, 10] (4)			

Numbers in parentheses show the total time steps that a system-generated PC-set occurs under the respective chart PC-set.

**TABLE 3:** System interpretations of chart chords for “Au Privave” without solo (top) and with random (bottom) solo at epoch 59, shown as pitch class sets.

No soloChart chord		System interpretations	
[0, 5, 9]	[2, 5, 10] (6)	[0, 5, 9] (61)	[0, 5, 9, 10] (12)	
[2, 5, 7, 10]	[2, 5, 7, 10] (60)	[0, 4, 7, 9] (36)	[2, 5, 9] (16)	
[0, 4, 7, 10]	[0, 4, 7, 10] (35)	[2, 5, 8, 10] (4)	[1, 3, 7, 10] (4)	[0, 2, 6, 9] (4)
[0, 3, 7, 10]	[0, 3, 7, 10] (16)			
[1, 3, 5, 9]	[0, 3, 5, 9] (1)	[0, 3, 6, 8] (2)	[5, 8, 11] (9)	
[2, 5, 8, 10]	[2, 5, 10] (1)	[2, 5, 8, 10] (28)	[3, 6, 10, 11] (3)	
[1, 5, 8, 10]	[1, 5, 8, 10] (16)			
[1, 3, 7, 10]	[2, 5, 8, 10] (16)			
[0, 4, 7, 9]	[2, 5, 7, 10] (8)	[0, 2, 5, 9] (4)	[0, 4, 7, 9] (4)	
[0, 2, 6, 9]	[0, 4, 5, 9] (12)	[2, 5, 9, 10] (4)	[0, 2, 5, 9] (4)	[0, 2, 6, 9] (12)
**Random solo** **Chart chord**		**System interpretations**	
[0, 5, 9]	[2, 5, 10] (3)	[0, 5, 9] (73)	[0, 5, 9, 10] (3)	
[2, 5, 7, 10]	[0, 5, 9] (1)	[2, 5, 7, 10] (83)	[0, 4, 7, 9] (12)	[2, 5, 9] (16)
[0, 4, 7, 10]	[0, 4, 7, 10] (46)	[0, 2, 6, 9] (1)		
[0, 3, 7, 10]	[0, 3, 7, 10] (16)			
[1, 3, 5, 9]	[0, 3, 5, 9] (5)	[0, 3, 6, 8] (6)	[2, 6, 9, 11] (1)	[2, 5, 9, 11] (2)
[2, 5, 8, 10]	[2, 5, 10] (1)	[2, 5, 8, 10] (31)		
[1, 5, 8, 10]	[1, 5, 8, 10] (16)			
[1, 3, 7, 10]	[2, 5, 8, 10] (4)	[1, 3, 7, 10] (12)		
[0, 4, 7, 9]	[0, 4, 7, 9] (16)			
[0, 2, 6, 9]	[0, 2, 5, 9] (2)	[0, 2, 6, 9] (30)		

Numbers in parentheses show the total time steps that a system-generated PC-set occurs under the respective chart PC-set.

**TABLE 4:** System interpretations of chart chords for “Au Privave” without solo (top) and with random (bottom) solo at epoch 1,251, shown as pitch class sets.

No solo Chart chord	System interpretations				
[0, 5, 9]	[1, 3, 10, 11] (5)	[0, 3, 5, 9] (5)	[0, 3, 7, 8] (1)	[3, 6, 11] (1)	[2, 6, 8, 11] (1)
	[1, 3, 7, 10] (1)	[0, 5, 8] (1)	[0, 3, 8] (1)	[2, 7, 11] (1)	[0, 5, 9] (51)
	[5, 8, 11] (4)	[2, 7, 10] (4)	[0, 5, 7] (3)		
[2, 5, 7, 10]	[2, 5, 8, 10] (2)	[2, 5, 7, 10] (59)	[0, 1, 5, 8] (1)	[2, 3, 7, 10] (1)	[0, 5, 8] (1)
	[1, 3, 10, 11] (4)	[1, 4, 6, 9] (4)	[0, 4, 7, 10] (10)	[1, 4, 7, 9] (4)	[0, 3, 7] (4)
	[2, 5, 10] (4)	[0, 2, 5, 7] (12)	[0, 2, 5, 9] (3)	[0, 2, 7, 10] (3)	
[0, 4, 7, 10]	[0, 3, 7] (16)	[2, 5, 10] (1)	[1, 3, 6, 10] (1)	[0, 4, 7, 10] (24)	
[0, 3, 7, 10]	[0, 3, 7, 10] (16)				
[1, 3, 5, 9]	[0, 3, 5, 9] (1)	[0, 1, 5, 8] (8)	[1, 3, 7, 10] (3)		
[2, 5, 8, 10]	[3, 5, 8, 11] (4)	[0, 3, 7] (7)	[2, 7, 10] (3)	[2, 5, 8, 10] (4)	[2, 5, 10] (7)
	[0, 5, 8] (4)	[2, 5, 9] (3)			
[1, 5, 8, 10]	[3, 5, 7, 10] (10)	[1, 3, 7, 10] (6)			
[1, 3, 7, 10]	[1, 3, 7, 10] (2)	[0, 3, 8] (4)	[0, 5, 8] (4)	[3, 7, 10] (6)	
[0, 4, 7, 9]	[0, 4, 7, 9] (16)				
[0, 2, 6, 9]	[0, 2, 6, 9] (12)	[0, 3, 7] (8)	[0, 5, 8] (4)	[0, 5, 7, 8] (4)	
**Random solo** **Chart chord**	**System interpretations**				
[0, 5, 9]	[1, 3, 10, 11] (4)	[2, 5, 7, 10] (5)	[3, 5, 8, 10] (1)	[1, 3, 7, 10] (1)	[1, 3, 5, 8] (1)
	[1, 5, 8] (1)	[3, 5, 7, 10] (2)	[2, 5, 10] (2)	[3, 6, 11] (6)	[0, 4, 5, 9] (11)
	[2, 5, 8, 10] (5)	[0, 3, 7] (2)	[0, 5, 9] (17)	[3, 7, 10] (4)	[4, 8, 11] (3)
	[0, 3, 5, 9] (3)	[3, 5, 8, 11] (2)	[0, 4, 7, 10] (2)	[0, 5, 8] (3)	[0, 3, 8] (1)
[2, 5, 7, 10]	[1, 3, 7, 10] (2)	[0, 3, 7] (1)	[0, 3, 8, 10] (1)	[2, 6, 8, 11] (1)	[2, 5, 7, 10] (57)
	[5, 6, 8, 11] (2)	[0, 3, 5, 9] (2)	[2, 5, 9] (7)	[1, 3, 10, 11] (4)	[1, 4, 6, 9] (3)
	[1, 5, 8, 10] (1)	[2, 3, 7, 10] (3)	[1, 4, 9, 11] (2)	[1, 4, 6, 10] (3)	[0, 5, 7, 9, 10] (1)
	[0, 5, 8, 10] (1)	[1, 4, 9] (1)	[0, 5, 9] (1)	[2, 5, 10] (4)	[5, 8, 11] (1)
	[0, 2, 3, 5, 10] (1)	[3, 6, 8, 11] (1)	[0, 2, 5, 9] (1)	[0, 3, 7, 8] (2)	[0, 5, 8] (1)
	[2, 7, 11] (1)	[2, 5, 9, 10] (1)			
[0, 4, 7, 10]	[0, 3, 7] (6)	[3, 5, 7, 8] (1)	[0, 5, 8] (2)	[0, 4, 7, 10] (18)	[0, 3, 8] (1)
	[2, 5, 10] (3)	[1, 4, 6, 9] (1)	[1, 3, 6, 10] (1)	[3, 6, 8, 11] (1)	[2, 7, 10] (1)
	[0, 3, 7, 8] (2)				
[0, 3, 7, 10]	[0, 3, 7, 10] (11)	[1, 4, 9, 11] (1)	[1, 3, 10, 11] (1)	[3, 7, 10] (2)	[0, 1, 5, 8] (1)
[1, 3, 5, 9]	[0, 3, 5, 9] (5)	[1, 4, 6, 10] (1)	[2, 6, 8, 11] (1)	[0, 3, 5, 8] (1)	[1, 3, 7, 10] (1)
	[0, 5, 8] (1)	[0, 3, 7] (3)			
[2, 5, 8, 10]	[2, 7, 10] (1)	[2, 5, 8, 10] (19)	[0, 5, 8] (5)	[1, 3, 7, 10] (1)	[3, 7, 10] (1)
	[2, 5, 10] (3)	[2, 5, 7, 10] (2)			
[1, 5, 8, 10]	[1, 3, 6, 10] (1)	[1, 3, 5, 10] (3)	[1, 3, 7, 10] (2)	[3, 5, 7, 10] (3)	[1, 5, 8, 10] (6)
	[2, 5, 8, 10] (1)				
[1, 3, 7, 10]	[1, 3, 7, 10] (8)	[0, 5, 8] (1)	[3, 6, 10] (1)	[3, 7, 10] (4)	[0, 3, 8] (1)
[0, 4, 7, 9]	[0, 4, 7, 9] (12)	[0, 2, 3, 5, 10] (1)	[2, 5, 7, 10] (1)	[1, 3, 5, 6, 8] (1)	
[0, 2, 6, 9]	[0, 2, 6, 9] (10)	[0, 3, 5, 9] (2)	[0, 3, 7] (5)	[0, 5, 8] (3)	[0, 2, 5, 9] (3)
	[2, 5, 7, 11] (1)	[0, 1, 3, 8] (1)			

Numbers in parentheses show the total time steps that a system-generated PC-set occurs under the respective chart PC-set.

**FIGURE 2:**
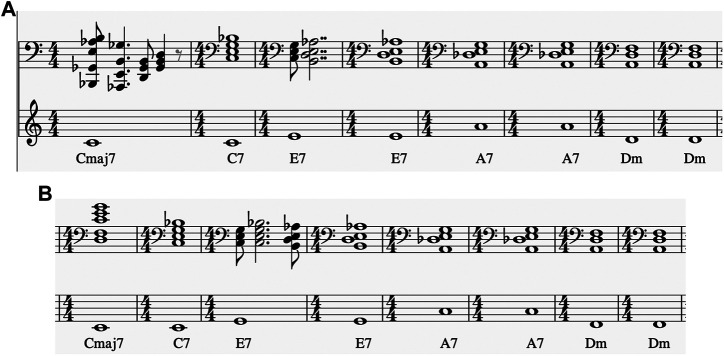
First eight measures **(A)** and measures 33–40 **(B)** of system-generated chords over the respective lead sheet chords for “All of Me” with random solo part (ommitted in the depiction).

Regrading “All of Me”, Table 1 shows that in most cases the exact harmonic description in the lead sheet chart is reflected by the system. Initially, it should be noted that harmonic deviations mostly concern the first few starting measures of each piece, where the system has not incorporated any memory in its decisions. The beginning chord of the chart, [0, 4, 7, 11], appears to have the most alterations, some of which are clearly erroneous (e.g. the [4, 6, 8, 10, 11] interpretation that was composed for random solo). Figure 2A shows the first eight measures and Figure 2B measures 33 to 40, composed by the system for “All of Me” in a real-time simulation setting with random solo (the solo part is not shown). The “erroneous” choices appear to be artefacts of the initial delay of the system to catch up with the constraints and start building up harmonic memory; Figure 2A shows that the first three chord shown in the lower part of Table 1 are a result of this delay. Other harmonic deviations concern the delay of the system in complying with “unexpected” chord changes – given that most pieces in the dataset are pop songs. For instance, some misinterpretations of the E7 chord ([2, 4, 8, 11]) are a result of delay in “comprehending” the unexpected change; this is shown in the third bars of both **(A)** and **(B)** parts of Figure 2. System-generated chords for “Au Privave” follow a similar pattern in terms of harmonic compliance but with fewer erroneous harmonic deviations, as evident in Table 3.

### Variability

The chords generated by the system in each improvisation setting for each piece are expected to be different, since different improvisations from the human soloist should trigger different responses. Those differences are examined by direct comparison of the system generated chords for the two improvisation modes, i.e. the chords generated by the system without human solo and with a random solo. A general figure that describes the differences between the system-generated chords in both examined pieces with (random) and without solo, is given by computing the percentage of chords that are different per time step for accompaniment sessions comprising four repetitions of the entire chart, with (random) and without solo. In “All of Me” only 2% of system-generated chords are different between random and no solo for epoch 59, which jumps to 60% for epoch 1,251, showing that the system decisions are affected slightly by the presence of a solo in early epochs, while the effect of solo is more evident as epochs progress. In “Au Privave” this percentage starts from 74% during epoch 59 and jumps to 84% at epoch 1,251, showing that system generations are more sensitive to the presence of a chord solo for this piece.

For observing the differences within each improvisation session, the system-generated chords in four repetitions of the entire chart are examined repetition-by-repetition – forming four quarters of the entire composition, referred to as “quartiles”. Tables 5–8 show the quartile similarities for “All of Me” (epochs 59 and 1,251) and “Au Privave” (epochs 59 and 1,251) respectively, without (left) and with random solo (right). In “All of Me” and with an absence of solo, both in the early and the late epoch of training only the first repetition is different from the remaining three, as show in the first rows and columns of both matrices in Tables 5, 7. The insertion of the random solo does not influence the overall result in the early epoch (right matrix in Table 5), but for the late epoch the influence is evident (right matrix in Table 7). Therefore, the example of “All of Me” shows that training the system for more epochs allows some sense of responsiveness to human input, as evident by the variability that emerged from the random solo. In “Au Privave”, on the other hand, the incorporation of the random solo (Table 6) influences each repetition even from early training epochs, therefore creating different variations of the chart in each of the four iterations (except repetition three and four that differ only by 1%); variations for this test piece are even more evident in the more progressed training epoch (Table 8).

**TABLE 5:** “Quartile” similarity in system-generated chords in “All of Me” without (left) and with random solo (right) at epoch 59.

	No solo			
	1st qrt	2nd qrt	3rd qrt	4th qrt
1st qrt	0.00	0.09	0.09	0.09
2nd qrt	0.09	0.00	0.00	0.00
3rd qrt	0.09	0.00	0.00	0.00
4th qrt	0.09	0.00	0.00	0.00

	Random solo			
	1st qrt	2nd qrt	3rd qrt	4th qrt
1st qrt	0.00	0.05	0.05	0.06
2nd qrt	0.05	0.00	0.00	0.00
3rd qrt	0.05	0.00	0.00	0.00
4th qrt	0.06	0.00	0.00	0.00

**TABLE 6:** “Quartile” similarity in system-generated chords in “Au Privave” without (left) and with random solo (right) at epoch 59.

	No solo			
	1st qrt	2nd qrt	3rd qrt	4th qrt
1st qrt	0.00	0.34	0.34	0.35
2nd qrt	0.34	0.00	0.00	0.01
3rd qrt	0.34	0.00	0.00	0.01
4th qrt	0.35	0.01	0.01	0.00

	**Random solo**			
	**1st qrt**	**2nd qrt**	**3rd qrt**	**4th qrt**
1st qrt	0.00	1.00	0.99	0.99
2nd qrt	1.00	0.00	0.33	0.34
3rd qrt	0.99	0.33	0.00	0.01
4th qrt	0.99	0.34	0.01	0.00

**TABLE 7:** “Quartile” similarity in system-generated chords in “All of Me” without (left) and with random solo (right) at epoch 1,251.

	No solo			
	1st qrt	2nd qrt	3rd qrt	4th qrt
1st qrt	0.00	0.53	0.53	0.54
2nd qrt	0.53	0.00	0.00	0.00
3rd qrt	0.53	0.00	0.00	0.00
4th qrt	0.54	0.00	0.00	0.00

	**Random solo**			
	**1st qrt**	**2nd qrt**	**3rd qrt**	**4th qrt**
1st qrt	0.00	0.51	0.72	0.19
2nd qrt	0.51	0.00	0.30	0.55
3rd qrt	0.72	0.30	0.00	0.72
4th qrt	0.19	0.55	0.72	0.00

**TABLE 8:** “Quartile” similarity in system-generated chords in “Au Privave” without (left) and with random solo (right) at epoch 1,251.

	No solo			
	1st qrt	2nd qrt	3rd qrt	4th qrt
1st qrt	0.00	0.40	0.40	0.41
2nd qrt	0.40	0.00	0.00	0.01
3rd qrt	0.40	0.00	0.00	0.01
4th qrt	0.41	0.01	0.01	0.00

	**Random solo**			
	**1st qrt**	**2nd qrt**	**3rd qrt**	**4th qrt**
1st qrt	0.00	0.65	0.96	0.70
2nd qrt	0.65	0.00	0.91	0.73
3rd qrt	0.96	0.91	0.00	0.83
4th qrt	0.70	0.73	0.83	0.00

**FIGURE 3:**
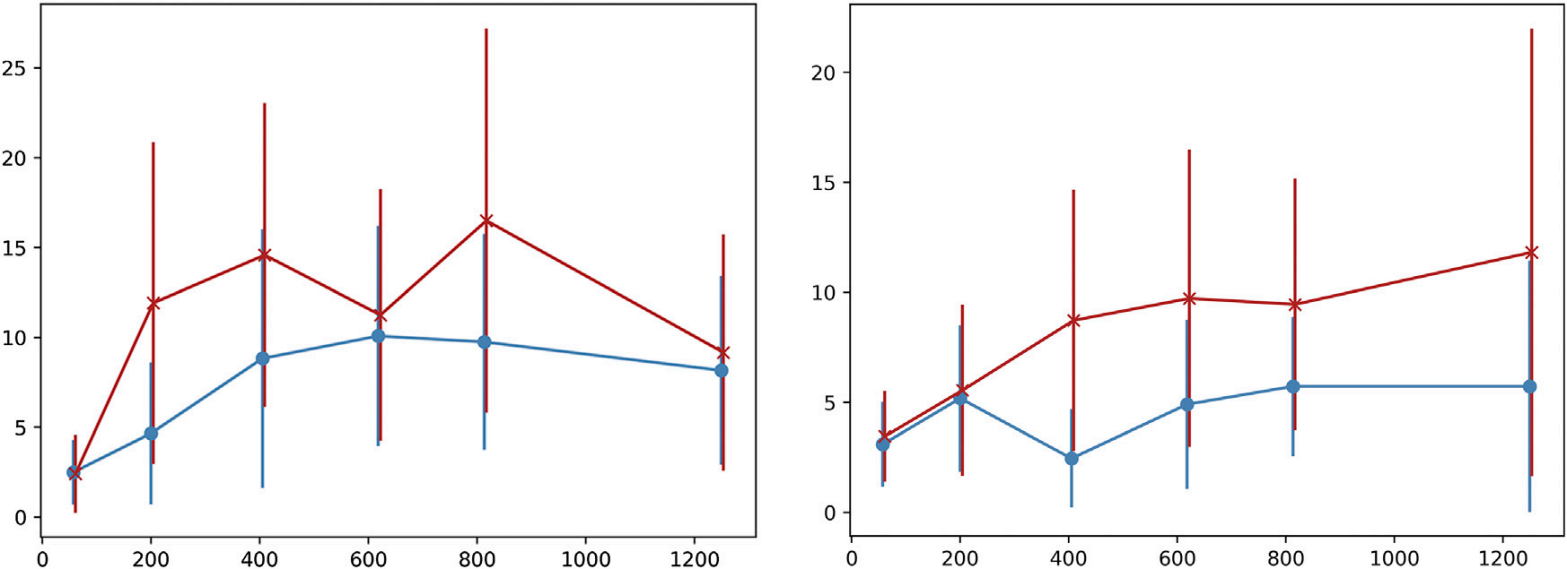
Error-bars of different voicings employed by the system for each chord label in the chart accross a sampled set of epochs.

**FIGURE 4:**
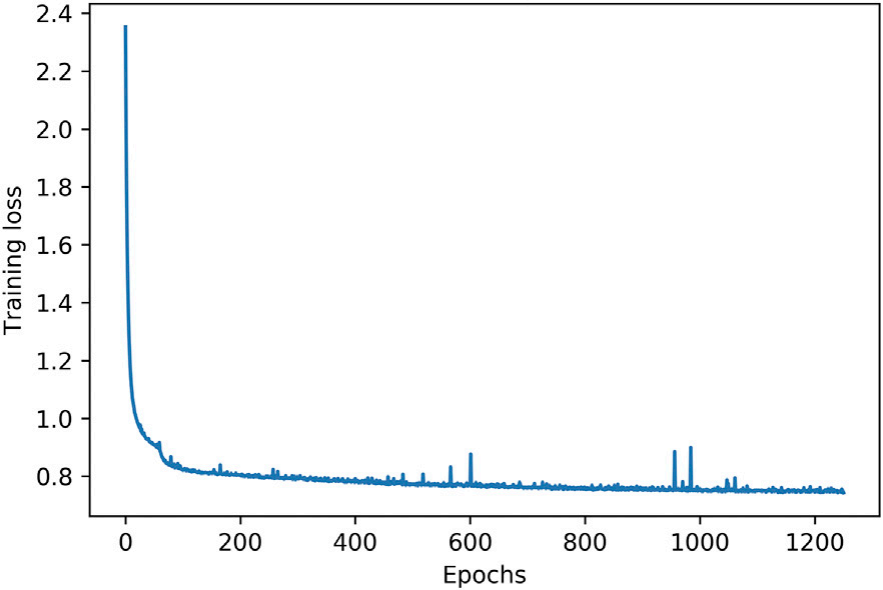
Loss of the training objective function in the validation set across multiple epochs.

A final examination of variability in the generated chords is performed by measuring the number of different voicings per chord symbol on the chart. This is a more detailed examination of how the PC-sets presented in Tables 1–4 are further split down in voicing layouts, i.e. what is the variability in terms of inversions and note doublings in the chords generated by the system. Figure 3 shows the average number of different voicings composed by the system for each chord label in the chart, in form of errorbars for some random epochs sampled accross all training epochs. In “All of Me” (left image), each chord symbol in the chart is materialised with approximately 2.5 different voicing implementations in epoch 59, almost regardless of the presence of solo (red “x” indicates presence of random melody and blue circle absence thereof). The system presents increased voicing variability dependence on human solo input for this piece as the epochs increase. In the case of “Au Privave,” the tendency of the system to become more dependent on human input becomes more evident as epochs increase. The error value of the objective function in a validation set during training is shown in Figure 4. The typical decrease that is observed indicates that there is a relation between error loss and system adaptability to human input, i.e., better training leads to further variability.

### Listening Tests

The dataset used to train the artificial agent, contains a broad variety of popular western music melodies with simulated accompaniments derived from an augmentation process. Furthermore, the quantitative metrics presented in the previous subsections are not capable to completely capture the perceptual quality and originality of the chord accompaniments generated by the proposed system. To this end, we carried out a subjective evaluation based on listening tests, aiming to study whether the generated accompaniments are comparable to the original chords existing in the dataset.

For preparing the listening tests we randomly selected 10 solo melodies along with their original accompaniments from the validation set. Then we used the 10 selected melodic parts to generate their corresponding chord accompaniments with the proposed artificial agent, thus ensuring that the system receives novel input, not wielded during training. Accordingly, each participant was presented with 10 tests; each test included three audio clips, starting only with the melodic part and followed by its combinations with the two accompaniments (original and generated), which are introduced in a random order so as to avoid any possible biases. The actual audio excerpts had a duration of around 30 s and looped for six times to reach 3 min. Then, the participants had to answer the following three questions for each accompaniment (six questions per test) in a Likert scale from 1 (low) to 5 (high):

- Q1: Evaluate the overall high-level structure of the accompaniment with respect to the introduced melody.
- Q2: Evaluate the harmonic compliance of the accompaniment with reference to popular western music.
- Q2: Evaluate the rhythmical compliance of the accompaniment with reference to popular western music.

In our study 21 participants were involved, 15 male and six female, with the majority being 20–40 years old. All of the participants were musicians with different levels of expertise, having at least intermediate knowledge of music theory. Consequently, we collected a total of 1,260 answers and the results are presented in Table 9. By inspecting only the mean values we can observe that the participants evaluated slightly better the original accompaniments in most questions. However in order to determine whether this preference is statistically important, we performed a Wilcoxon rank sum test, having as null hypothesis that there is no difference between the two accompaniments. The calculated *p*-values demonstrated that there is statistically significant difference between the original and the generated accompaniments in examples 5, 6, 7, 9 and 10 (highlighted with bold fonts in Table 9), while we cannot reject the null hypothesis for the remaining examples. In other words, in 50% of the examples, we cannot be certain about whether the generated music is inferior to the original, as far as the examined qualities can define.

**TABLE 9:** Results of our listening tests.

	Question	*p*-value	Accompaniment	Median	Mean		Question	*p*-value	Accompaniment	Median	Mean
Example 1	Q1	0.48121	Original	4.0	3.57	Example 6	Q1	**0.04689**	Original	4.0	3.71
			Generated	3.0	3.33				Generated	3.0	2.9
	Q2	0.35198	Original	3.0	3.43		Q2	**0.00025**	Original	4.0	3.95
			Generated	4.0	3.71				Generated	2.0	2.48
	Q3	0.6966	Original	4.0	3.95		Q3	**0.00826**	Original	4.0	3.86
			Generated	4.0	3.86				Generated	3.0	2.86
Example 2	Q1	0.30236	Original	4.0	3.57	Example 7	Q1	**0.00007**	Original	4.0	4.19
			Generated	3.0	3.24				Generated	2.0	2.43
	Q2	0.44293	Original	4.0	3.52		Q2	**0.0**	Original	4.0	4.19
			Generated	3.0	3.24				Generated	1.0	1.62
	Q3	0.66891	Original	4.0	3.52		Q3	**0.00005**	Original	5.0	4.33
			Generated	4.0	3.33				Generated	2.0	2.43
Example 3	Q1	0.26296	Original	4.0	3.67	Example 8	Q1	0.48907	Original	4.0	3.95
			Generated	3.0	3.29				Generated	4.0	3.86
	Q2	0.08951	Original	4.0	3.86		Q2	0.1159	Original	4.0	4.24
			Generated	3.0	3.24				Generated	4.0	3.76
	Q3	0.95987	Original	4.0	3.71		Q3	0.88003	Original	4.0	3.9
			Generated	4.0	3.71				Generated	4.0	4.0
Example 4	Q1	0.32656	Original	4.0	3.86	Example 9	Q1	**0.03353**	Original	4.0	3.76
			Generated	4.0	3.52				Generated	3.0	3.0
	Q2	0.15523	Original	4.0	3.86		Q2	**0.00001**	Original	5.0	4.24
			Generated	3.0	3.38				Generated	2.0	2.24
	Q3	0.41361	Original	4.0	3.86		Q3	**0.00037**	Original	4.0	4.1
			Generated	4.0	3.52				Generated	2.0	2.57
Example 5	Q1	**0.0543**	Original	4.0	3.71	Example 10	Q1	**0.00153**	Original	4.0	3.95
			Generated	3.0	2.9				Generated	2.0	2.76
	Q2	**0.00022**	Original	4.0	3.76		Q2	**0.0014**	Original	4.0	3.71
			Generated	2.0	2.19				Generated	2.0	2.29
	Q3	**0.00766**	Original	4.0	4.0		Q3	**0.02863**	Original	4.0	3.52
			Generated	3.0	2.86				Generated	2.0	2.62

The bold fonts indicate the statistically significant differences provided by a Wilcoxon rank sum test between the original and the generated accompaniments.

Overall, we can say that the accompaniments generated by the proposed artificial agent had better rhythmical compliance rather than harmonic, which might be due to the metric information that is included in the system input. Also, the poor performance in some examples indicates that the computational capabilities of a single LSTM layer are limited, thus suggesting more sophisticated architectures to be tested. We strongly encourage the reader to visit the online repository and listen to the audio files of the listening tests.

## Conclusion

The paper at hand presented a study on how deep neural network architectures can be employed for simulating a jazz improvisation setting between a human soloist and an artificial accompanist, based on a common chord chart. A basic implementation incorporating deep neural networks was presented and publicly available data were transformed in a way that all necessary information for the task at hand became available, i.e. information about metric structure, lead sheet chords, human-generated solo/melody and system-generated accompaniment responses. The motivation of this work is based on modeling the interplay between expectation and its violation by two improvising musicians (one human and one artificial) with implicit machine learning approaches (deep neural networks) and the methodology included the development of “a model within a model”, that allows the artificial agent to have its own model of expectation for the human improviser. Additional challenges included the adaptation of large amounts of data to the desired form, leading to the development of a data enrichment process that generated variability in the accompaniment parts of the collected pieces.

Results were obtained by testing the system in two real-time simulation settings: without any assumed human solo and with the inclusion of a random solo. The responses of the system under these two settings in two well-known jazz standards (“All of me” and “Au Privave”) indicated that harmonic compliance with the chart chords was mainly achieved, except mainly from the beginning of each accompaniment session where the system needs to “collect memory” for starting performing better; this is possibly due to the random initialisation of the states in the LSTM networks that are in the core of the presented basic implementation. Even though it was expected for the system to be influenced by the incorporation of a human solo, this was not the case in both examined pieces. Specifically, in “All of Me” the inclusion of a random solo did not appear to affect the output of the system, while the system-generated chords exhibited self-repetition in accompaniment sessions incorporating four iterations of the chart. Conversely, in “Au Privave” the inclusion of the random solo affected the system output both by decreasing self-repetition in four iterations and by increasing the number of chord voicings employed by the system for given chart chords. In order to evaluate the perceptual quality of the generated chords, we also performed a subjective evaluation based on listening tests, where participants had to compare original and generated accompaniments given their corresponding melodies, by ranking their harmonic and rhythmical compliance in a liker scale. A Wilcoxon rank sum test on the responses showed that 50% of the examples were not significantly inferior to the original accompaniments.

Future research is necessary for a more thorough examination of such system for real-time accompaniment. The results presented herein indicate that it is possible to model expectation and violation thereof for real-time jazz accompaniment with deep neural networks, however, severe limitations have to be acknowledged for performing further studies:

1. There is no proper data available with all the necessary information (lead sheet chords, metric information, solo and accompaniment). A crucial part of the data, i.e. the accompaniment, was actually constructed algorithmically while the solo part included melodies (rather than solos) with restricted expressional variability. The data enrichment method that was developed to construct artificial variability in the data was based on a rudimentary probabilistic implementation which is not enough for creating consistent connections that could be learned from the system.
2. The execution time of predictions might be marginally acceptable for scalable real-time systems. For the presented study, time resolution was significantly reduced for making the system safely compatible with real-time conditions, however, this fact reduced the expressional capabilities of the system. This includes not only restricted capabilities for the system responses, but also restricted capabilities for the system to identify expressional characteristics of the human soloist.
3. The prominent style found in the dataset was pop, which comprises smaller harmonic variability in comparison to jazz. Therefore, the resulting accompaniment had to be creatively adjusted for more reflecting complex jazz lead sheet progressions. A consistent dataset of jazz standard accompaniment sessions is necessary for studying this problem more deeply.

## Data Availability

The datasets [generated/analyzed] for this study can be found in the Zenodo open-access repository on the link https://zenodo.org/record/3523222. The training code of the LSTM models, the real-time web interface and the audio files of the listening tests can be found on GitHub following the link https://github.com/kosmasK/JazzICat.

## Footnotes

1
https://www.cs.hmc.edu/∼keller/jazz/improvisor/ – last accessed February 1st, 2020.

2
https://github.com/wayne391/lead-sheet-dataset/ – last accessed February 1st, 2020.

3
https://web.mit.edu/music21 – last accessed February 1st, 2020.

4
https://github.com/kosmasK/JazzICat.
